# Assessment of Radiation Dose to Adult Patients Undergoing Cardiac CT

**DOI:** 10.7759/cureus.97386

**Published:** 2025-11-20

**Authors:** Asna Pheroz, Hana Khan, Areeba Khan, Mehtab Ahmad, Mudasir A Shah

**Affiliations:** 1 Medicine, Jawaharlal Nehru Medical College, Aligarh, IND; 2 Medicine, Royal Lancaster Infirmary, Lancaster, GBR; 3 Radiodiagnosis, Jawaharlal Nehru Medical College, Aligarh, IND

**Keywords:** bmi, cardiac ct, clinical audit, contrast media, ct angiography, ctca protocol, diagnostic reference levels, dose length product, effective dose, linear regression analysis

## Abstract

Background: This study is designed to evaluate the radiation doses received by adult patients during cardiac computed tomography (CT) examinations.

Materials and methods: A retrospective study was conducted at a tertiary care hospital from May 2024 to August 2024. Patients aged over 20 years undergoing CT angiography on a GE Healthcare Revolution EVO CT scanner following the standard protocol were included. Dose length product (DLP) values were generated by the scanner software for each patient, and the effective dose (E) was subsequently computed. Linear regression analysis was used to assess the relationship between effective dose, body mass index (BMI), and injected contrast volume.

Results: The mean value of effective dose was observed to be 13.55 mSv, with a minimum of value of 0.175 mSv and a maximum of 27.23 mSv. The median DLP was found to be 943.26 mGy.cm, with a minimum value of 12.47 mGy.cm. The linear regression analysis shows that there exists no dependence of effective dose on BMI and volume of the contrast injected.

Conclusion: The effective dose mean was 13.55 mSv, with a DLP median of 943.26 mGy.cm. Our audit shows that the mean values of "E" surpass the United Kingdom National Diagnostic Reference Levels (NDRLs) for coronary CT angiography and coronary angiography, 4.3 mSv and 170 mGy.cm, respectively. These findings highlight the need for dose optimization in cardiac CT protocols.

## Introduction

Heart disease is the primary cause of mortality in both men and women of all races and ethnic groups [1]. In recent times, there has been a steady rise in the proportion of young patients presenting with atherosclerotic cardiovascular diseases (ASVD) [2]. Furthermore, a growing global burden of coronary artery diseases (CAD) has become the leading cause of morbidity and mortality worldwide [3]. Congenital heart illnesses are among the major cardiac pathologies that have been linked to higher adult mortality, especially in young people [4]. In contrast, myocardial infarction, arrhythmias, and even rapid death can result from massive coronary artery aneurysms if they become occluded [5]. After myocardial infarction and stroke, pulmonary embolism ranks third in terms of vascular-related deaths and is a leading preventable cause of mortality for hospitalized patients [6].

Over the past 20 years, there have been significant technological advancements in computed tomography coronary angiography (CTCA) [7]. Its high sensitivity and specificity for identifying coronary heart disease account for its increased usage in the evaluation of stable chest discomfort [8,9]. Although CTCA is a very powerful diagnostic tool, it necessitates ionizing radiation exposure and an intravenous contrast material injection [10]. Several strategies have been adopted to minimize this radiation exposure. At the hardware level, efforts are ongoing to create more efficient detectors for CT units. In terms of image acquisition, new techniques have been introduced to restrict exposure to smaller segments of the cardiac cycle. Lastly, advancements in image reconstruction technology aim to enhance the signal-to-noise ratio in CT images while using fewer photons in the raw data [10]. Several attempts have been made to record the radiation doses in CTCA. Kalamar et al. [11] conducted a retrospective radiation dose audit and suggested that radiation doses might be reduced without sacrificing the quality of diagnostic images. Despite the implementation of various initiatives to reduce radiation exposure, there is a need to assess the radiation exposure levels with CT examination [12].

Although CTCA is being used more clinically in India, there remains a dearth of practice-based, region-specific data on radiation doses received by patients. The majority of research assessing CT radiation exposure comes from developed nations with standardized procedures and dose-reduction tools, which could not accurately represent the conditions in Indian medical systems. A study conducted in the UK showed that the radiation burden sustained by CTCA can be reduced significantly without compromising image quality by using dose-saving algorithms [13]. Radiation exposure may be impacted by the various scanner technologies used in Indian imaging centers, the fluctuating patient demographics, and the uneven application of protocols. Furthermore, there are presently no national diagnostic reference levels (NDRLs) specifically designed for cardiac CT in India, which hinders attempts to evaluate and optimize dosages for patient safety. Furthermore, the extent to which Indian radiology teams employ dose-reduction techniques is not well-documented. To enhance clinical practice and establish local diagnostic reference levels (LDRLs), these gaps must be filled. This audit aims to assess the radiation doses received by adult patients undergoing cardiac CT examinations and to compare the observed values with the NDRLs established in the United Kingdom.

## Materials and methods

Study design

This retrospective study was conducted in a tertiary care hospital from May 2024 to August 2024. The Institutional Ethics Committee of the university gave its approval to this retrospective study (Ref. No: IEC JNMC/1344 dated: 11.05.2024). Informed consent was waived due to the retrospective nature of the study and the use of anonymized data, which involved no additional risk or financial burden to the patients. 

Study sample

The following simple formula was used for calculating the adequate sample size in prevalence:

$n= \frac{Z^{2}P(1-p)}{d^{2}}$

where n is the sample size, Z is the statistic corresponding to level of confidence, P is expected prevalence (that can be obtained from same studies or a pilot study conducted by the researchers), and d is precision (corresponding to effect size). The minimum sample size calculated for our audit study was 50 patients.

Inclusion Criteria

Adult patients above the age of 20 years undergoing CT Angio on GE Healthcare Revolution EVO CT, which is installed in the Department of Radiodiagnosis in accordance with standard protocol.

Exclusion Criteria

Patients below the age of 20, those who were unable to cooperate during imaging, and those who had any medical conditions that would prevent them from receiving CT imaging were excluded from the study.

Image Acquisition

All patients had their CT scans performed according to a standard procedure, which included the following restrictions: head towards the CT gantry spine position. For image acquisition, the appropriate scanning range was chosen. The CT exams were conducted using a CT unit that is installed in our department. The CT unit used the Filtered Back Projection (FBP) algorithm for image reconstruction, and the preset imaging limits were as follows: tube voltage [kVp (kilovoltage peak): 80-120], quality reference (mAs: 100), detector collimation (128×0.6 mm), acquisition matrix (512×512), field-of-view [(314×314 mm)], slice acquisition thickness (5 mm), and inter-slice spacing (1 mm). The patient's identity was kept confidential in all 192 patients, and the data were analyzed anonymously.

The aforementioned CT unit underwent routine Quality Assurance (QA) testing to confirm the appropriateness and quality of patient care. These tests were part of the QA program: slice thickness/radiation profile width (a), operational potential measurement (b), timer accuracy (c), mAs linearity measurement (d), output consistency (e), Computed Tomography Dose Index measurement (f), low resolution of contrast (g), high contrast resolution (h), levels of radiation leakage from the X-ray tube housing (i), and the installation's radiation protection survey (j). The operator handbook was followed for scanning the phantom, and the outcomes of each component were compared to the ideal values. The tests were conducted with slice thicknesses of 10 mm and 5 mm, scan settings of 120 kVp, 100 mAs, and 3 seconds. All of the outcomes fell within the tolerance range that the manufacturer, and the Atomic Energy Regulatory Board (AERB), Government of India, had recommended.

Effective Dose

According to ICRP report-60 [14,15], the weighted average of organ dose values H_T_ for a number of designated organs is the effective dose (E):

$E = \sum_{i} w_{i} H_{t,i}$ (1)

"E" is measured in millisieverts (mSv). Each organ's tissue weighting factor (w_i_) reflects how sensitive it is to radiation-induced effects, which establishes how much of "E" that organ contributes. The effective dose cannot be determined in vivo. Anthropomorphic phantom-based thermoluminescent dosimeter (TLD)-based measurements are not the best for daily usage due to their lengthy processing times. Therefore, "E" is determined by multiplying the dose length product (DLP) (mGy.cm) by the age and site compensated conversion factor (K). Consequently, "E":

 $E = K \times DLP$ (2)

For various body areas and (standard) patient age groups, the normalized effective dose per DLP values is represented by "K," the conversion factor. Specifically, the conversion factor "K" used in this study was 0.026 mSv/mGy·cm, corresponding to the chest region for adult patients, based on the normalized effective dose per DLP values provided by Huda and Atherton and Shahid et al. [16-18]. Following every CT imaging study, the DLP (mGy.cm) is shown as a dosage sheet, from which the value "E" is calculated. The CT unit’s teamplay software (syngo.via software version B40) automatically recorded and displayed DLP (mGy.cm), Volume Computed Tomography Dose Index (CTDIvol), and total mAs for each patient-specific scan in the dose report. The DLP is a measure of the total radiation output during the scan, combining dose per slice with scan length.

Body Mass Index

Each patient's height and weight were measured before imaging, and a particular, calibrated tool (Indosurgicals: weight and height measurement apparatus) was used to calculate their body mass index (BMI). The BMI data subcategories were used to categorize the patients as follows: underweight was defined as $\text{BMI} &lt; 18.5~\mathrm{kg/m^2}$; normal weight as $18.5 \leq \text{BMI} &lt; 24.9~\mathrm{kg/m^2}$; overweight as $25 \leq \text{BMI} &lt; 29.9~\mathrm{kg/m^2}$; and obese as $\text{BMI} \geq 30~\mathrm{kg/m^2}$ [19].

Statistical analysis

The statistical analysis was performed using Origin 6.0 (v6.1052 [B232], OriginLab Corporation, Northampton, MA 01060, USA) software. The descriptive statistics, including mean, median, minimum, maximum, and standard deviation, were computed for age, BMI, DLP, effective dose, and volume of contrast injected. A powerful technique of linear regression analysis for predicting the value of a dependent variable by modeling its connection with one or more independent variables was employed to investigate the dependence of effective dose (E) on BMI (kg/m^2^) and dependence of effective dose (E) on volume of contrast injected (ml). 

## Results

**Figure 1:**
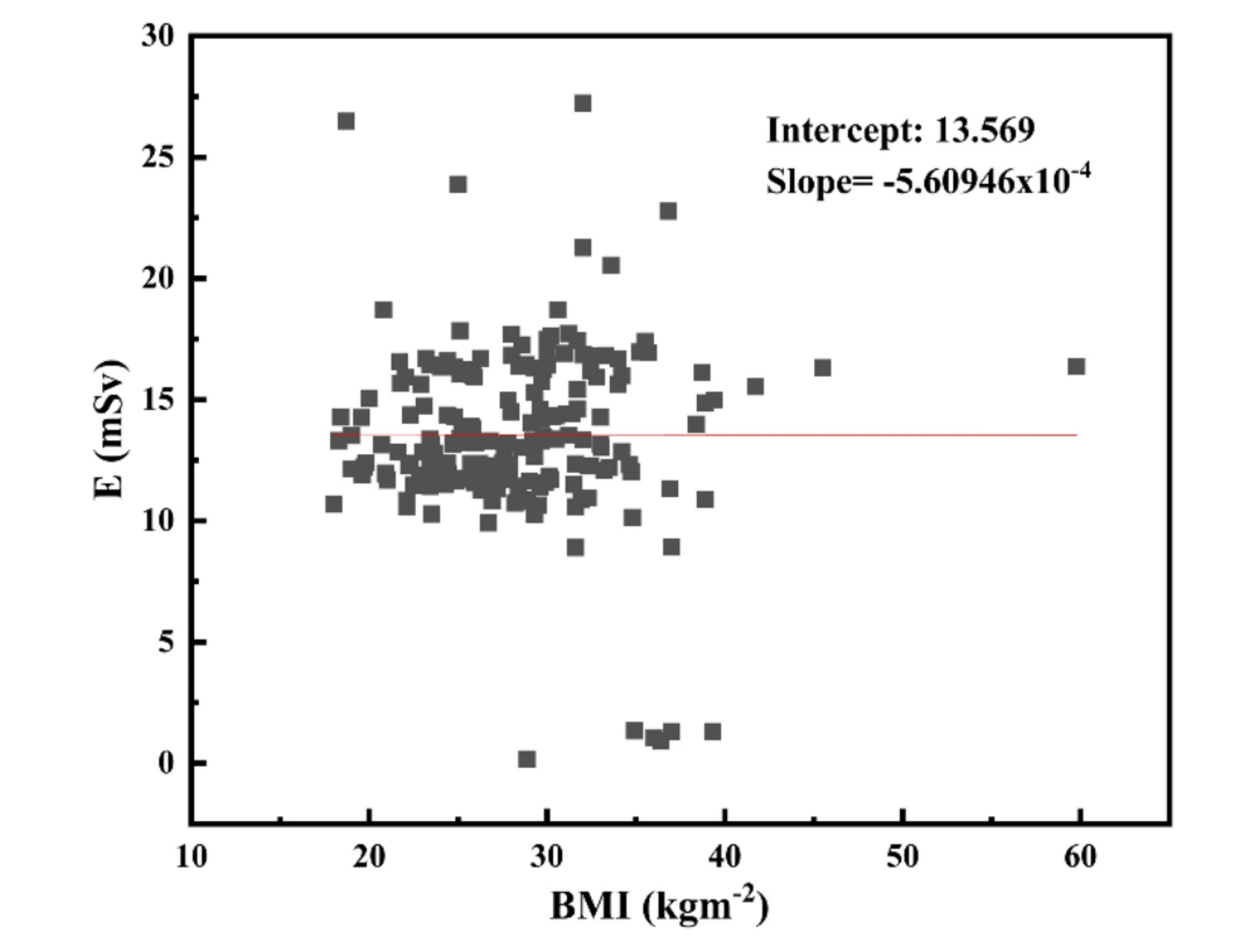
Correlation between effective dose (E) and BMI. BMI: body mass index.

**Figure 2:**
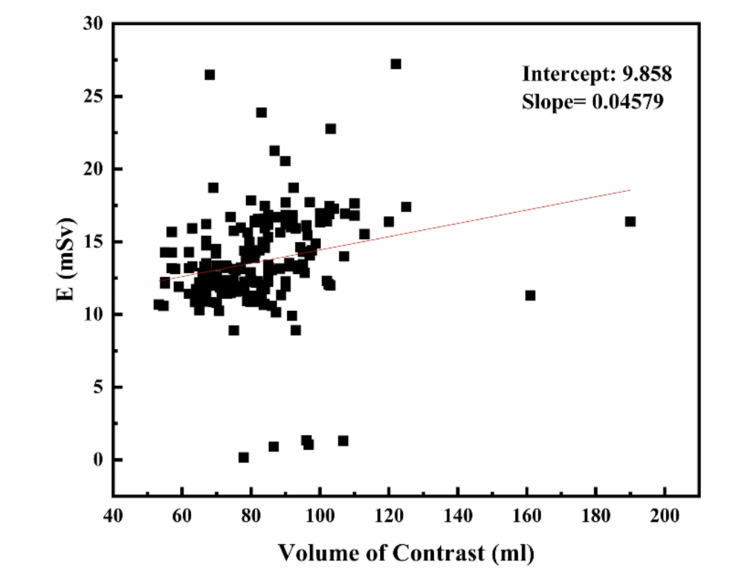
Correlation between effective dose (E) and volume of the contrast injected.

A total of 192 adults (male=105 and female=87), with an average age of 48.34 years, ranging from 21 to 81 years, were retrospectively included in the study for the estimation of effective dose. Based on BMI classification, 18 patients (9.37%) had high risk obesity, 49 (25.52%) were obese, 68 (35.42%) were overweight, 56 (29.17%) had normal BMI, and only 1 (0.52%) was underweight. The statistical analysis for E, BMI, age, DLP, and volume of the contrast injected is presented in Table 1. The mean value of effective dose was observed to be 13.55 mSv, with a minimum value of 0.175 mSv and maximum of 27.23 mSv. The median DLP was found to be 943.26 mGy.cm, with a minimum value of 12.47 mGy.cm. The linear regression analysis was performed to investigate the dependence of effective dose on BMI and volume of the contrast injected. As observed from the linear regression analysis (Figures 1, 2), there is no dependence of effective dose on BMI and volume of the contrast injected. 

**Table 1:** Statistical analysis of the effective dose, DLP (mGy.cm) and BMI (kg/m²). BMI: body mass index; DLP: dose length product.

Parameter	Age (years)	BMI (kg/m²)	DLP (mGy.cm)	E (mSv)	Volume of contrast injected (ml)
Mean	48.34	28.21	968.0	13.55	82.13
Std. Dev.	11.04	5.62	257.09	3.60	16.86
Median	48	27.9	943.26	13.20	80.7
Min	22	18.0	12.47	0.175	53.3
Max.	81	59.78	1945.44	27.23	190

On comparing the DLP and effective dose of our study with the NDRLs for the UK, it is found that the values of DLP and effective dose are much higher than the NDRLs for CTCA and coronary angiography.

## Discussion

Patients are exposed to a comparatively higher radiation dose during CT imaging. Radiation damage to a single cell's deoxyribonucleic acid (DNA) can serve as the precursor to a modified cell that is still able to divide. Despite the body's generally effective defenses, there is a chance that this type of radiobiological effect, heightened by the stimulation of other mediators not necessarily connected to the biological impact initiated by ionizing radiation, could cause cancer. If the primary damage is to the germ cells of the gonads, genetic effects could also arise. At low doses, the probability of radiation-related stochastic effects increases with dosage and is proportional to the absorbed dose. The chance frequently increases with dosage, rather than in a simple proportion, at higher doses and dose rates. Even if a single examination raises a patient's chance of developing cancer by a small amount, in developed countries, the typical person has one of these examinations annually; as a result, the overall risk rises dramatically. Globally, the number of CT examinations is increasing, and as a result, more emphasis is being placed on the various types of clinical investigations that utilize CT. However, the rapid advancements in tomography imaging did not allow for the development of strategies to reduce the patient doses required for any particular imaging operation, which is contrary to the general trend in diagnostic radiology. As a result, controlling the patient's dosage during CT imaging is essential, and creating LDRLs in the form of dose restrictions is crucial to ensuring that radiation safety precautions are properly taken [14,17,18].

Due to the three different radiation dosage characteristics, the radiation dose distribution in CT differs greatly from that of traditional radiography procedures. First, the highly collimated nature of the primary X-ray beam reduces the volume of tissue exposed to radiation from the beam during the acquisition of a single CT image. Second, the irradiated tissue volume is exposed to the x-ray beam from almost every angle during the rotating acquisition, which more evenly distributes the radiation dosage to the tissues in the beam. Finally, high contrast resolution in CT acquisition requires a high signal-to-noise ratio (SNR), which greatly enhances the radiation dose to the slice because of the higher kilovoltage (kV) and milliampere-seconds (mAs) approaches used. Additionally, a considerable quantity of radiation is dispersed, sometimes surpassing the radiation dosage from the main beam. Significant dosage from scatter is given to surrounding tissues outside of the primary beam during the collection of a CT slice because scattered radiation is not limited to the collimated beam profile like primary X-rays [17]. Depending on the machine settings, the radiation dose for a single CT scan of the organ being examined can vary from 15 mSv in adults to 30 mSv in newborns. For each investigation, two to three CT scans are usually conducted. At these levels, the most likely (although small) danger is radiation-induced carcinogenesis. Because of their innate radiosensitivity and the longer time range over which radiation-induced cancer is more likely to appear, children are at a higher risk from radiation than adults, making the worry even more pressing [20]. The use of ECG-gated acquisition in patients with congenital heart failure (CHD) is the first issue; the best methodology is the second. Each technique should have its radiation dosage evaluated in order to minimize radiation exposure [17]. As a result, controlling patient dosage during CT imaging is essential to guaranteeing that radiation safety measures are taken as directed [21].

Diagnostic reference levels (DRLs) are a technique used to optimize ionizing radiation-based medical imaging processes. They provide an estimate of the anticipated radiation dose that a patient of average size might receive during a specific imaging technique. Imaging facilities might find operations that might be open to further optimization by comparing the typical (median) exposure levels for popular imaging procedures with DRLs. The National Diagnostic Reference Level Service was developed by a number of countries, including the Australian Radiation Protection and Nuclear Safety Agency, to help imaging facilities compare their usual doses with the national DRLs and to make data collection easier for the establishment of national DRLs in Australia. National DRLs have been set for image-guided and interventional procedures, nuclear medicine, and computed tomography. To make sure they represent current practice, DRLs must be continuously reviewed and revised by the national authority. This continuous cycle of evaluation and review aids in maximizing the benefit-to-risk ratio for patients [22].

Such audits serve as a baseline for defining the LDRLs for an institution and a country, and this audit report is crucial for evaluating the dosage to the patient during cardiac CT. The aim of our audit is to assess the radiation doses received by the adult patients during cardiac CT examination and to compare our findings with the NDRLs for the UK. The DLP and "E" are excellent measures of radiation dose from CT and could be used to immediately improve the radiation safety standards by identifying when doses are much higher than the reference values. The mean value of effective dose was observed to be 13.55 mSv, with a minimum value of 0.175 mSv and a maximum of 27.23 mSv. The median DLP was found to be 943.26 mGy.cm, with a minimum value of 53.3 mGy.cm. The linear regression analysis was performed to investigate the dependence of effective dose on BMI and the volume of the contrast injected. As observed from the linear regression analysis (Figures 1, 2), there is no dependence of effective dose on BMI and the volume of the contrast injected. The poor correlation between E versus BMI and E versus volume of the contrast injected may be attributed to the patient ethnicity. As the premier tertiary care referral center in Western Uttar Pradesh, India, our hospital, Central University Medical College, serves patients from all socioeconomic backgrounds and is located within a 150 km radius. Additionally, many international students, faculty members, and students from other regions of India come in for imaging. The DLP and effective dose of our study is higher than the NDRLs for the UK [23]. It is found that the values of DLP and effective dose for our study are much higher than the NDRLs for CTCA and coronary angiography (170 mGy.cm for DLP and 4.3 mSv for effective doses). 

The report has a few shortcomings like being a single centric study and only one CT unit being used for imaging. This report is from a Central University Medical College, which is tertiary care referral center working as the apex center in Western Uttar Pradesh, India, with the patients of all strata presenting from a radius of 100-150 km.

## Conclusions

The mean value of effective dose was observed to be 13.55 mSv, with a minimum value of 0.175 mSv and a maximum of 27.23 mSv. The median DLP was found to be 943.26 mGy.cm, with a minimum value of 12.47 mGy.cm. The effective dose and DLP from our audit surpass the UK's NDRLs. It is discovered that the effective dose and DLP values are significantly more than the NDRLs for CTCA and coronary angiography, which are 4.3 mSv for effective doses and 170 mGy.cm for DLP. The results of the study suggest that we need to tailor the CTCA protocol for each patient to reduce the effective dose without compromising image quality. Further, it is recommended from the audit report to optimize CT protocols by adjusting radiation dosage based on patient size and clinical needs to reduce exposure without compromising diagnostic accuracy. Unnecessary radiation can be reduced by better patient selection through improved identification of those in need of CT angiography and considering non-radiation diagnostic options. Promoting public awareness can improve safer imaging by educating about radiation risks and exposure limits. The results of this study emphasize the importance of conducting regular audits of radiation doses and regularly comparing institutional doses with domestic and international benchmarks.
